# Prevalence of Multimorbidity in Lithuania: Insights from National Health Insurance Fund Data

**DOI:** 10.3390/jcdd12020047

**Published:** 2025-01-26

**Authors:** Dovilė Ramanauskaitė, Roma Puronaitė, Audronė Jakaitienė, Sigita Glaveckaitė

**Affiliations:** 1 Clinic of Cardiac and Vascular Diseases, Institute of Clinical Medicine, Faculty of Medicine, Vilnius University, 03101 Vilnius, Lithuania; roma.puronaite@santa.lt (R.P.); sigita.glaveckaite@santa.lt (S.G.); 2 Center of Cardiology and Angiology, Vilnius University Hospital Santaros Klinikos, 08661 Vilnius, Lithuania; 3 Institute of Data Science and Digital Technologies, Faculty of Mathematics and Informatics, Vilnius University, 08412 Vilnius, Lithuania

**Keywords:** multimorbidity, chronic diseases, healthcare resources, cardiac multimorbidity

## Abstract

(1) Background: As the burden of multimorbidity is increasing worldwide, little is known about its prevalence in Lithuania. We aimed to estimate the prevalence of chronic conditions and multimorbidity among Lithuanian adults and assess their impact on healthcare utilization. (2) Methods: A retrospective analysis of the Lithuanian National Health Insurance Fund database was performed in 2019. Multimorbidity was defined as having two or more chronic conditions. (3) Results: Of the Lithuanian population, 1,193,668 (51.5%) had at least one chronic condition, and 717,386 (31.0%) had multimorbidity. Complex multimorbidity (CM) was present in 670,312 (28.9%) patients, with 85.0% having complex cardiac multimorbidity (CCM) and 15.0% having complex non-cardiac multimorbidity (CNM). Multimorbidity increased with age, from 2% at age 18–24 to 77.5% at age 80 and above, and was more prevalent among women (63.3% vs. 36.7%, *p* < 0.001). One-third of multimorbid patients were hospitalized at least once per year, with half staying for a week or longer. CCM patients were more likely to be hospitalized, rehospitalized, and have more primary care visits (OR: 2.23, 1.60, 4.24, respectively, all *p* < 0.001). (4) Conclusions: Multimorbidity in Lithuania increases with age and affects women more. Chronic cardiovascular diseases contribute to a higher prevalence of multimorbidity and a more significant burden on the healthcare system.

## 1. Introduction

The World Health Organization (WHO) defines multimorbidity as the presence of at least two chronic diseases in one patient [1]. As the population ages, the occurrence of more than one chronic condition continues to rise, making multimorbidity a common phenomenon [2,3,4]. The prevalence of multimorbidity varies in the literature, depending on its definition, study designs, and patient characteristics [2,3]. The estimated pooled prevalence in meta-analyses ranges from 33.1% to 42.4%, with reported rates showing very high heterogeneity (ranging from 2.7% to 95.6%) [2,3,5]. Multimorbidity affects patients’ quality of life and mental well-being, leads to disability, and increases mortality rates [2,3,6]. It also places a burden on the healthcare system by increasing the number of outpatient visits, hospitalizations, and overall healthcare costs [3,5].

In the context of multimorbidity, cardiovascular diseases become increasingly important, as they are age-related and often diagnosed along with other diseases [7]. Approximately two-thirds of patients with cardiovascular disease are diagnosed with another chronic condition from the age of 70 [8,9]. The risk of developing a second or third cardiovascular disease is significantly higher than the risk of developing the first one [7]. The European Society of Cardiology highlights that multimorbid cardiovascular patients have been underrepresented in most clinical trials from which the guidelines have been derived and encourages the analysis of multimorbidity using registries and big data [8].

As the prevalence of chronic diseases grows, the single-disease-focused healthcare model becomes ineffective. Estimating the prevalence of chronic non-communicable diseases is essential to identify target population groups for intervention, optimize management strategies, and enable clinicians to provide high-quality care. The global heterogeneity in multimorbidity prevalence limits the use of this information as a universal standard across countries. There is a lack of research on this topic in Lithuania, with only one article published so far evaluating the prevalence of chronic diseases in 2014 [10]. As multimorbidity has increased over the past few decades, analyzing more recently available data on multimorbidity in Lithuania is essential.

We aimed: (1) to estimate the prevalence of chronic conditions and multimorbidity among Lithuanian adults in 2019, (2) to assess the prevalence of complex multimorbidity (CM), including complex cardiac multimorbidity (CCM), and (3) to evaluate the impact of complex cardiac and non-cardiac multimorbidity (CNM) on the utilization of healthcare resources.

## 2. Materials and Methods

### 2.1. Study Database

We performed a retrospective cohort study that analyzed the Lithuanian National Health Insurance Fund (NHIF) database. Since Lithuania mandates compulsory health insurance, the NHIF database contains a substantial part of the Lithuanian electronic health records. In 2018, the database consisted of 98% of the healthcare information of Lithuania’s population [11]. The data under analysis covers 1 January 2019 to 31 December 2019. These records include patients’ demographic data and disease diagnosis, as well as information about outpatient (primary care and specialist) and inpatient (hospitalizations) healthcare visits. All visits are linked to diagnosis codes from the International Statistical Classification of Diseases and Related Health Problems, Tenth Revision, and Australian Modification (ICD-10-AM).

The anonymous use of the NHIF database for research purposes was approved by the Biomedical Research Ethics Committee of the Vilnius Region (approval number: 2020/3-1207-692).

### 2.2. Study Cohort

We included adults aged 18 and older covered by Lithuanian mandatory health insurance in 2019 with at least one chronic disease from the list (Table 1). The patients’ age was determined based on their age in 2019.

We identified patients as multimorbid according to the WHO’s accepted definition, which defines multimorbidity as two or more chronic diseases [1].

Since there is no universally accepted list of chronic conditions, we selected diseases according to Barnett et al. [6]. Barnett et al. highlighted the increasing prevalence of multimorbidity and its strong association with age and socioeconomic deprivation. The outlined set of diseases included hypertension, diabetes, coronary heart disease, depression, chronic obstructive pulmonary disease, and other conditions that form the core for analyzing multimorbidity, covering various medical specialties and having been previously used in other studies [10].

To evaluate the impact of cardiovascular diseases on the prevalence of multimorbidity, we categorized patients according to the complexity of multimorbidity: complex multimorbidity, complex cardiac multimorbidity, and complex non-cardiac multimorbidity. CM was defined as having two or more chronic diseases from at least two different medical specialties from the list (Table 1). CCM was defined as having two or more chronic diseases from at least two specialties, with cardiology as one of them. CNM included patients with two or more chronic diseases from at least two specialties, excluding cardiology.

The hospitalizations, length of stay, rehospitalizations (defined as hospital readmission within a period of up to 30 days), and outpatient visits (primary and specialists) were chosen to evaluate healthcare system utilization.

### 2.3. Statistical Analysis

Quantitative variables were reported as either the median with interquartile ranges or the mean with standard deviation (SD), depending on which better reflected the data’s variability. Categorical variables were presented as frequencies and percentages. The Pearson’s chi-square, Mann–Whitney U, and Kruskal–Wallis tests were used for group comparisons.

Given that the healthcare utilization outcomes were non-normally distributed variables with excessive zeros (indicating a large number of persons not using healthcare resources), two-part hurdle models were used to assess the association between multimorbidity or the number of chronic conditions and healthcare service utilization (e.g., the annual frequency of hospitalizations per person or the number of specialist visits per person per year). Hurdle models consist of two regression equations: the probability of observing non-zero outcomes (logit model) and the count of non-zero outcomes (negative binomial regression). The truncated negative binomial models were used where zero values were impossible—such as the total annual length of stay for hospitalized individuals.

The results of the hurdle models were presented as odds ratios (OR), which indicate the relationship between multimorbidity or the number of chronic conditions and the likelihood of having healthcare utilization, as well as incidence rate ratios (IRR), which reflect the change in the rate of healthcare utilization associated with multimorbidity or with the number of chronic conditions. IRRs were reported for truncated models, as these models do not include zero counts.

The population prevalence of chronic diseases was calculated using variables from official demographic data provided by the State Data Agency (Statistics Lithuania) at the Lithuanian Open Data Portal [12]. Statistical analysis was performed using R (version 4.4.1) [13], packages pscl [14,15].

The results were considered statistically significant if the *p*-value was less than 0.05.

## 3. Results

### 3.1. Study Cohort Characteristics

In 2019, the study database included 1,193,668 patients with at least one chronic condition, representing 51.5% of all individuals registered in Lithuania at that time, according to data from the State Data Agency (Table 2). The average patient’s age was 60 ± 16 years. The majority of the Lithuanian population were women, who also had a higher prevalence of at least one chronic condition (723,784 (60.6%) women vs. 469,884 (39.4%) men, *p* < 0.001).

### 3.2. The Prevalence of Chronic Conditions and Their Impact on Healthcare Utilization

**Figure 1:**
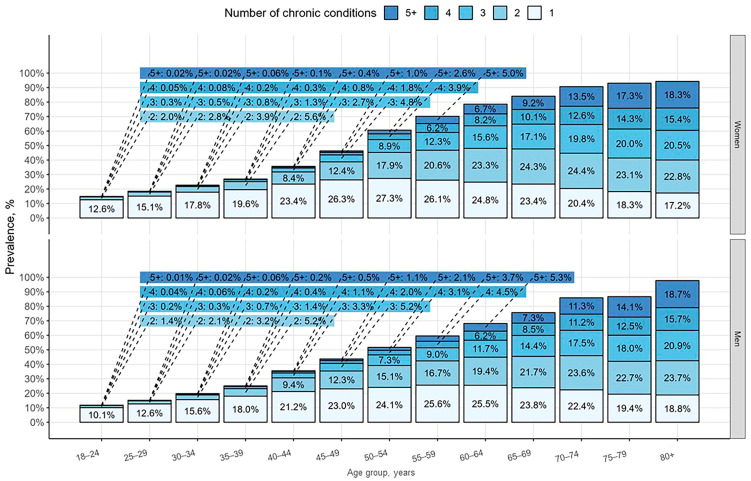
The prevalence and number of chronic conditions by age and gender groups in the general population.

The number of patients with one chronic disease increased with age, varying from 13.3% in the 18–24 age group to 95.2% among individuals 80 years old and older (*p* < 0.001). The prevalence and number of chronic conditions based on gender and different age groups are shown in Figure 1.

When patients were grouped based on the number of chronic conditions, 39.9% had one chronic condition, while only 8.8% were diagnosed with five or more chronic conditions (*p* < 0.001) (Table 3). The proportion of women increases from 56.7% to 67.4%, while men decrease from 43.3% to 32.6% as the number of diseases rises (from one to five and more) (*p* < 0.001). Patients with more chronic diseases were older, with an average age of 71 ± 11 years among those with five or more chronic conditions (*p* < 0.001).

Healthcare resource utilization analysis showed that a quarter of the study population (296,237 (24.8%)) was hospitalized at least once during the year, and 50.7% of these hospitalizations lasted longer than a week (Table 3). Comparing patients with different numbers of diseases, patients with five or more conditions had the most prolonged hospital stays (14.62 ± 15.34, *p* < 0.001), and a greater proportion of these patients required more than seven bed days (39,408 (70.4%), *p* < 0.001). An increase in the number of conditions was associated with more patients requiring rehospitalizations (13,001 (23.2%) patients with five or more diseases vs. 7809 (10.4%) patients with one disease, *p* < 0.001). The numbers of specialist visits were also more frequent in five and more chronic diseases group compared to one (15.30 ± 9.80 vs. 6.74 ± 5.62; 12.23 ± 9.81 vs. 4.32 ± 5.25, respectively, all *p* < 0.001).

Regression models were used to comprehensively analyze healthcare utilization across groups with different numbers of chronic diseases (Table S1). The odds of having any hospitalization, rehospitalization, or specialist visits increased with the number of chronic conditions. Patients with five or more diseases were 5.84 times more likely (95% CI: 5.75, 5.93, *p* < 0.001) to be hospitalized and had 2.89 (95% CI: 2.82, 2.97, *p* < 0.001) times higher hospitalization rate compared with the reference group (patients having one chronic disease). Patients with five or more diseases also had 2.04 times longer (95% CI: 2.00, 2.08, *p* < 0.001) hospitalizations. Compared to patients with one chronic condition, those with two, three, four, and five or more conditions were from 1.15 to 2.58 times more likely to be rehospitalized (*p* < 0.001). Furthermore, patients with five or more diseases were 7 times more likely (95% CI: 6.42, 7.64, *p* < 0.001) to have primary healthcare visits and 9.79 times more likely (95% CI: 9.52, 10.1, *p* < 0.001) to have specialist visits.

### 3.3. The Prevalence of Multimorbidity and Its Impact on Healthcare Utilization

In the Lithuanian population, 717,386 patients (31.0%) had multimorbidity, and 670,312 (28.9%) were classified as having CM (Table 4). Among patients with CM, 85.0% had CCM, while 15.0% presented with CNM. The mean age of patients with two or more chronic conditions was 65 ± 14 years. Multimorbidity was more prevalent among women than men (453,785 (63.3%) vs. 263,601 (36.7%). A higher proportion of women than men were diagnosed with both CCM and CNM (362,274 (63.6%) vs. 207,458 (36.4%) and 66,219 (65.8%) vs. 34,361 (34.2%), respectively, *p* < 0.001). A significantly higher age was observed among multimorbid patients with cardiovascular diseases (67 ± 13 years in CCM vs. 55 ± 15 in CNM, *p* < 0.001).

**Figure 2:**
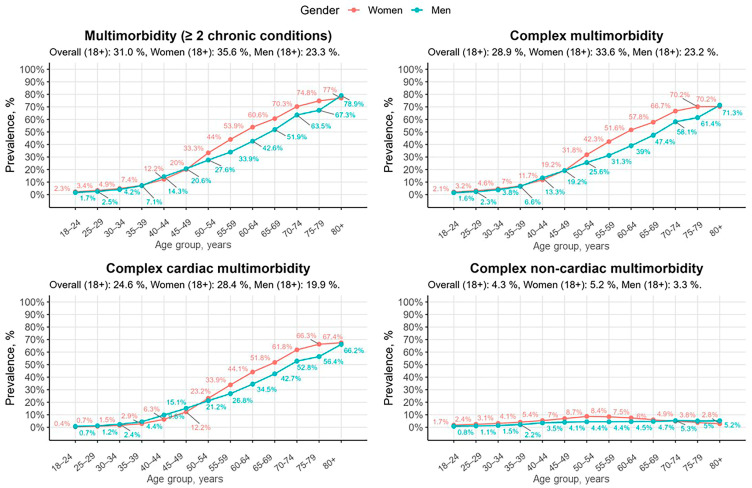
Prevalence of multimorbidity in the Lithuanian population by age groups and gender.

The prevalence of multimorbidity in the Lithuanian population by age group and gender is shown in Figure 2. As illustrated, women consistently demonstrate higher prevalence rates of chronic conditions compared to men across all age groups and multimorbidity types. The disparity between women and men was particularly pronounced between the ages of 55 and 79 across all multimorbidity groups, except CNM. The prevalence of CNM was less age-dependent than that of CCM.

The prevalence of multimorbidity increased significantly with age, starting from 2.0% in the youngest age group (18–24 years) and peaking at 77.5% in individuals aged 80 years and older. In contrast, the prevalence of individuals with only one chronic condition peaked in middle-aged adults (55–59 years at 25.9%) and declined in the older population. A shift between one chronic condition and multimorbidity occurred around the age of 50–54, where multimorbidity surpassed one chronic condition, becoming the dominant pattern in older populations.

The utilization of healthcare resources was also assessed among multimorbidity groups (Table 4 and Table 5). It was revealed that one-third of multimorbid patients (221,017 (30.8%)) experienced at least one hospitalization per year, while half of them (123,729 (56.0%)) were hospitalized for a week or longer. When comparing CCM and CNM, the number of patients hospitalized per year was significantly higher in the CCM group (180,881 (31.7%) vs. 27,199 (27.0%), *p* < 0.001).

Interestingly, within the CCM group, more patients were hospitalized for a week or longer (104,205 (57.6%) vs. 12,825 (47.2%), *p* < 0.001), whereas in the CNM group, more patients had 1–3 bed days per year (48,205 (26.7%) vs. 10,143 (37.3%), *p* < 0.001). Applying hurdle models to five outcomes revealed that both cardiac and non-cardiac multimorbidity groups showed significantly increased healthcare utilization compared to individuals with a single disease. The CCM patients demonstrated slightly higher hospitalization odds (OR 2.23 vs. 1.96) and frequency rates (IRR 1.75 vs. 1.67) compared to CNM. The CCM patients were 1.6 times more likely to be rehospitalized, with a 12% higher rehospitalization rate compared with CNM patients. In contrast, although CNM patients had slightly lower odds of rehospitalization (1.47 times more likely), their rehospitalization rates were significantly higher, increasing by 56.0%. In addition, the CCM group had higher odds (OR 4.24, 95% CI: 4.09, 4.39) of having primary healthcare visits and a higher number of visits rate (IRR 1.75, 95% CI: 1.75, 1.76). Specialist visits were 5.89 times more likely (95% CI: 5.73, 6.05) and 1.94 times more frequent (95% CI: 1.92, 1.95) among non-cardio patients.

## 4. Discussion

We conducted a retrospective Lithuanian population cohort study to evaluate the prevalence of chronic conditions and multimorbidity and to assess their burden on the healthcare system using data from the Lithuanian NHIF database. The study concluded that: (1) in Lithuania, 51.5% of patients have at least one chronic disease, and 31.0% suffer from multimorbidity; (2) multimorbidity increases with age, is more prevalent among women and is associated with increased likelihood of hospitalizations, rehospitalizations and higher amount of primary care visits than a reference group with single chronic disease.

### 4.1. The Rising Prevalence of Multiple Chronic Conditions

Evaluating the prevalence of chronic conditions is essential for a greater understanding of patients with long-term illnesses in the ageing world. This knowledge enhances patient management, reduces mortality, optimizes healthcare organization, and lowers healthcare costs [2,3]. Until now, in Lithuania, only one study has assessed the prevalence of chronic diseases, covering the period from January 2012 to June 2014 [10]. In that study, the overall multimorbidity prevalence was 16.3%; in contrast, our study found a significantly higher prevalence of 31.0%. This difference may indicate rising rates of chronic diseases in Lithuania, aligning with global trends. Due to advances in medical care and better survival rates, the global rise in multimorbidity has been observed since 2000 and stabilized between 2011 and 2021 [3]. Despite the increasing prevalence of multimorbidity, higher levels of disease accumulation remain relatively uncommon. We found that the proportion of affected patients decreases as the number of diseases rises. In our study, 26.1% of patients had two chronic diseases, while only a small proportion had five or more (8.8%). Similar results were reported by Pefoyo et al., where 12.3% of patients were diagnosed with two chronic diseases vs. 2.7% of patients with five or more [16].

Comparing multimorbidity prevalence with other studies is challenging due to methodological differences. However, comparisons can be made with global research that summarizes the literature. A recent systematic review and meta-analysis by Chowdhury et al. found an overall prevalence of multimorbidity of 37.2% [3]. Our study identified a slightly lower prevalence rate. We found that 31.0% of Lithuanians in 2019 had at least two conditions of 31 chronic diseases listed for the study. This difference may result from coding practices in our healthcare system, which prioritizes recording only those conditions that require active treatment, further testing, or specialist care. A higher prevalence of chronic diseases is observed in regions with private health insurance and payment systems depending on the diagnosis code [5]. Furthermore, our multimorbidity cohort was older than in Chowdhury’s study, with an average age of 56.95 ± 10.85 years compared to 65 ± 14 years in our cohort. As age is a well-established risk factor for multimorbidity, the lower prevalence of chronic diseases in the older age cohort may be attributed to unrecording. Nguyen et al. conducted a systematic review and meta-analysis and reported a pooled prevalence of multimorbidity at 33.1% [2]. In this study, the prevalence of multimorbidity was more similar to our findings. However, they included a more significant proportion of studies from low- and middle-income countries, associated with lower prevalence rates. For the high-income countries, the overall prevalence of 37.9% was slightly higher than what we observed. While, in comparison with regional studies, a similar prevalence of multimorbidity has also been reported in Estonia, where rates of at least one chronic condition were 49.1%, and two and more were 30.1% [17].

It is well known that the prevalence of multimorbidity varies by age and gender [2,3,4,5,6,10,18,19,20]. In line with the literature, we found that chronic diseases increased with age [21]. Middle-aged adults tend to visit hospitals more frequently, while older adults are challenged to access healthcare facilities regularly due to cognitive decline and physical limitations [22]. They also often present with overlapping or atypical symptoms. These barriers complicate the diagnostic process and delay timely treatment in older age groups [23,24]. Similar to findings in the literature, we observed that by age 65, more than half of our study population had at least two chronic conditions [2,17], while 80% of patients had at least one chronic condition [16]. Considering that individuals aged 65 and older account for about one-fifth of the population in Europe, this highlights the substantial healthcare burden posed by chronic diseases [25].

According to the literature, chronic conditions are noticeably more common among women [3,18,19,23,26]. This trend was observed in our study, as women showed a higher prevalence of at least one chronic condition (60.6% vs. 39.4%, *p* < 0.001), as well as multimorbidity (63.3% vs. 36.7% *p* < 0.001). It is suggested that the prevalence of chronic diseases among women is influenced by risk factors such as low physical activity and being overweight [27]. However, it is well known that women are more likely to seek medical help and report health issues [21,28,29]. We found that as the number of diseases increases (from one to five and more) the proportion of women grows from 56.7% to 67.4%. Since the life expectancy among Lithuanian women is higher (in 2019, 81 years vs. 71.5 years in men) [30], this may also reflect a more active role of women in using healthcare resources, leading to a higher number of chronic diseases.

### 4.2. Cardiovascular Diseases Impact on Prevalence of Multimorbidity

Our study showed that the prevalence of CCM is higher than CNM. We found that 85.0% of patients had multimorbidity involving at least one cardiovascular condition. Heart diseases have been identified as one of the most common initial diagnoses among patients who subsequently develop multimorbidity [29,31]. Moreover, studies investigating the structure of multimorbidity define hypertension as a predominant condition [4,16,32,33,34]. Since cardiovascular diseases often develop at an early age, and cardiology patients are more likely to seek active treatment by visiting healthcare specialists and participating in preventive programs, this results in a higher number of additional diagnoses [18]. Cardiovascular diseases remain the leading cause of morbidity and mortality worldwide [35]. It is evidenced that the development of cardiovascular disease in multimorbid patients leads to poorer prognosis [36,37]. Lawson et al., using data from 10,575 patients in the Swedish Heart Failure Registry, found that non-cardiovascular comorbidities were associated with much higher overall symptom burdens and more severe symptoms than cardiovascular comorbidities [38]. The literature analyses cardiovascular multimorbidity (with at least two cardiovascular diseases) [36], whereas we analyzed CCM (with one cardiovascular and another non-cardiac condition), demonstrating that even a single cardiovascular disease can contribute to a higher prevalence of multimorbidity. While two or more cardiovascular diseases facilitate disease management due to similar pathophysiological mechanisms, clinical decisions become more complicated with complex multimorbidity [16].

We also observed gender differences in CCM. In our cohort, more women than men were attributed to this multimorbidity subgroup (63.6% women vs. 36.4% men). Similarly, Tisminetzky et al. found that women were more prevalent in mixed multimorbidity groups (≥2 cardiovascular and ≥1 non-cardiovascular comorbidities) compared to men [39]. Increasing evidence highlights gender disparities in cardiovascular diseases. Studies show that cardiovascular diseases tend to develop 7–10 years later in women than in men, making women more vulnerable [40]. The first diagnosis of cardiovascular multimorbidity for women occurs at an average age of 73 [41]. In our study, patients with CCM were older than those in other multimorbidity groups, with a higher proportion of women in this group (multimorbidity 65 ± 14 years; CCM 67 ± 13 years; CNM 55 ± 15 years). This shows that older women not only face a growing prevalence of chronic conditions but also an increasing burden of cardiovascular diseases [42,43]. Identifying and targeting this multimorbidity subgroup’s prevention strategies could significantly improve patient care. A more detailed analysis of the structure of complex cardiac multimorbidity should be a focus of future research.

### 4.3. Healthcare Utilization

Reducing the burden associated with multiple chronic conditions remains a healthcare priority. It is well known that having more than one chronic disease increases the use of healthcare resources [18,21,39,44,45]. Our study revealed that more chronic conditions were associated with more frequent hospitalizations and rehospitalizations, extended hospital stays, and more primary and specialist visits. Furthermore, the odds of having any healthcare services increased with the number of chronic conditions. Buja et al. found that having more chronic diseases leads to a greater number of hospital admissions and a higher likelihood of spending more days in hospital per year [44]. In the study by Jankovic et al., more non-communicable chronic conditions were associated with a higher use of healthcare resources [19]. In contrast, in the study by Zhong et al., multimorbidity was the influencing factor [21].

It is suggested that not only the number of diseases but also the pattern may influence healthcare utilization [46]. Our study highlighted that multimorbidity with cardiovascular diseases is associated with a higher burden on the healthcare system. We found that the CCM group used more healthcare services such as hospitalizations and rehospitalizations and had longer hospital stays and more primary care visits. The specialist visits were more frequent among CNM patients. As patients with cardiovascular conditions experience more frequent disease exacerbations, accompanied by more prolonged and complicated hospitalizations, this may result in a lower number of specialist visits. This supports a study comparing multimorbidity phenotypes among patients with heart failure [47]. The study demonstrated that different multimorbidity patterns influence healthcare facility use, with the ‘malignant’ type of multimorbidity being linked to more prolonged hospitalizations and unplanned readmissions compared to less ‘malignant’ multimorbidity. Furthermore, it has already been shown that patients with myocardial infarction and non-cardiac comorbidities were more likely to have prolonged hospital stays compared to those without any non-cardiac comorbidities [37]. In addition, Canivell et al. revealed that multimorbid patients with myocardial infarction and non-cardiac conditions have a higher risk of experiencing recurrent cardiovascular events, with a rate of 12.78 per 100 person-years, compared to patients with only cardiovascular conditions, who have a rate of 7.79 per 100 person-years [36]. However, in that study, the highest risk was observed in patients with myocardial infarction and both cardiac and non-cardiac conditions with a rate of 20.45 per 100 person-years, indicating that multiple cardiovascular diseases along with non-cardiac conditions lead to poorer outcomes. As we see, the cardiovascular multimorbidity pattern leads to increasing healthcare needs. Therefore, in an ageing population with multimorbidity, disease management strategies should focus not only on single conditions but also on addressing coexisting comorbidities to optimize healthcare utilization.

### 4.4. Study Limitations

This study has some limitations. Our research used health administrative data, which covers a large part of the Lithuanian population and enables population-based analysis. However, the use of administrative data presents certain challenges. Firstly, coding practices may affect the data quality, resulting in overestimated or unreported conditions. Secondly, administrative data lack subjective information, such as patient status, type, and severity of symptoms. Lastly, the one-year study period limits the ability to observe long-term disease prevalence trends.

Additionally, multimorbidity research lacks a standardized methodology, a uniform multimorbidity definition, and a standard list of chronic conditions, which limits the ability to compare findings with other studies. Furthermore, we did not analyze the structure of multimorbidity to identify the high-burden conditions, which could be a focus of future research.

## 5. Conclusions

The prevalence of chronic conditions and multimorbidity in Lithuania is relatively high compared to global rates. It increases with age and is higher among women. As the population ages, the increase in multimorbidity leads to greater utilization of healthcare services. Cardiovascular diseases contribute to a higher prevalence of multimorbidity as well as a more significant burden on the healthcare system. Understanding the risks posed by rising rates of multiple chronic conditions is the crucial first step toward reducing their impact on the healthcare system and related expenditures.

## Figures and Tables

**Table 1:** The list of chronic conditions with ICD-10-AM diagnostic codes.

Chronic Condition	ICD-10-AM ^1^ Diagnostic Codes	Speciality
Cancer	C00–C96	Oncology
Anaemia	D50	Haematology
Hypothyroidism	E02, E03, E89.0	Endocrinology
Diabetes	E10.0–E10.9, E11.01–E11.9	Endocrinology
Obesity	E66	Endocrinology
Dyslipidemia	E78	Endocrinology
Dementia	F00.0–F00.9, G30.0–G30.9; F01.0–F01.9; F02.0–F02.8; F03	Neurology
Mental disorders	F20.0–F20.9, F30.0–F39, F40.00–F40.9, F41.0–F51.9, F42.0–F42.9, F43.0–F43.9	Psychiatry
Parkinson disease	G20	Neurology
Multiple sclerosis	G35	Neurology
Epilepsy	G40.00–G40.91	Neurology
Sleep apnoea	G47.3	Neurology
Back pain	G54.1, G54.4, G55.1; M51	Neurology
Glaucoma	H40-H42	Ophthalmology
Blindness	H53-H54	Ophthalmology
Hearing loss	H90.0–H90.8; H91.0–H91.9	Otolaryngology
Hypertension	I10–I15	Cardiology
Ischemic heart disease	I20–I25	Cardiology
Heart failure	I50.0–I50.9	Cardiology
Intracranial bleeding	I60–I62	Neurology
Stroke	I63–I64; I69	Neurology
Chronic obstructive oulmonary disease	J44.0–J44.9, J96	Pulmonology
Asthma	J45.0–J45.9	Pulmonology
Inflammatory bowel disease	K50–K51	Gastroenterology
Psoriasis	L40.0–L40.9	Dermatology
Rheumatoid arthritis	M05–M06	Rheumatology
Gout	M10.0–M10.99	Rheumatology
Osteoarthritis	M15–M19	Rheumatology
Systemic lupus erythematosus	M32	Rheumatology
Osteoporosis	M80–M82	Rheumatology
Renal failure	N18–N19	Nephrology

^1^ ICD-10-AM, International Statistical Classification of Diseases and Related Health Problems, Tenth Revision, and Australian Modification.

**Table 2:** The prevalence of at least one chronic disease by gender and age groups in the general population.

Characteristics	All	Age Groups												
		18–24	25–29	30–34	35–39	40–44	45–49	50–54	55–59	60–64	65–69	70–74	75–79	80+
General population *, *n* (%)	2,315,914 (100)	214,923 (9.3)	179,708 (7.8)	182,722 (7.9)	177,297 (7.7)	183,599 (7.9)	203,762 (8.8)	208,090 (9.0)	226,832 (9.8)	191,641 (8.3)	158,515 (6.8)	119,948 (5.2)	115,436 (5.0)	153,441 (6.6)
Prevalence of at least one chronic condition ^1,#^, *n* (%)	1,193,668 (51.5)	28,567 (13.3)	30,197 (16.8)	38,788 (21.2)	46,186 (26.1)	65,374 (35.6)	91,758 (45.0)	117,300 (56.4)	148,100 (65.3)	141,850 (74.0)	127,883 (80.7)	106,652 (88.9)	104,983 (90.9)	146,030 (95.2)
Median (Q1–Q3) of chronic conditions	2 (1–3)	1 (1–1)	1 (1–1)	1 (1–1)	1 (1–2)	1 (1–2)	1 (1–2)	2 (1–2)	2 (1–3)	2 (1–3)	2 (1–3)	2 (2–4)	3 (2–4)	3 (2–4)
Female population ^#^, *n* (%)	1,275,019 (55.1)	105,463 (49.1)	88,809 (49.4)	90,534 (49.5)	89,351 (50.4)	94,323 (51.4)	105,521 (51.8)	108,911 (52.3)	122,364 (53.9)	107,443 (56.1)	94,409 (59.6)	76,586 (63.8)	77,086 (66.8)	114,219 (74.4)
Prevalence of women with at least one chronic condition ^1,†^, *n* (%)	723,784 (56.8)	15,681 (14.9)	16,407 (18.5)	20,564 (22.7)	24,073 (26.9)	33,625 (35.6)	48,852 (46.3)	66,047 (60.6)	85,885 (70.2)	84,458 (78.6)	79,359 (84.1)	69,413 (90.6)	71,734 (93.1)	107,686 (94.3)
Male population ^#^, *n* (%)	1,040,895 (44.9)	109,460 (50.9)	90,899 (50.6)	92,188 (50.5)	87,946 (49.6)	89,276 (48.6)	98,241 (48.2)	99,179 (47.7)	104,468 (46.1)	84,198 (43.9)	64,106 (40.4)	43,362 (36.2)	38,350 (33.2)	39,222 (25.6)
Prevalence of men with at least one chronic condition ^1,$^, *n* (%)	469,884 (45.1)	12,886 (11.8)	13,790 (15.2)	18,224 (19.8)	22,113 (25.1)	31,749 (35.6)	42,906 (43.7)	51,253 (51.7)	62,215 (59.6)	57,392 (68.2)	48,524 (75.7)	37,239 (85.9)	33,249 (86.7)	38,344 (97.8)

All *p*-values are less than 0.001. Test used to compare patients across age groups: ^1^ Pearson’s Chi-squared test. Percentages are based on: *—total population of 2,315,914 persons; ^#^—total population within each age group; ^†^—number of women within each age group; ^$^—number of men within each age group. Continuous variables are presented as mean ± standard deviation or median (interquartile range). Categorical variables are expressed as *n* (%).

**Table 3:** Characteristics and healthcare resource utilization based on the number of chronic conditions.

Characteristics		All	Number of Chronic Conditions				
			1	2	3	4	5+
Number of patients, *n* (%)		1,193,668 (100)	476,282 (39.9)	311,818 (26.1)	190,771 (16.0)	109,447 (9.2)	105,350 (8.8)
Gender ^1^, *n* (%)	Women	723,784 (60.6)	269,999 (56.7)	189,386 (60.7)	121,681 (63.8)	71,692 (65.5)	71,026 (67.4)
	Men	469,884 (39.4)	206,283 (43.3)	122,432 (39.3)	69,090 (36.2)	37,755 (34.5)	34,324 (32.6)
Age (year) ^2^, mean ± SD		60 ± 16	52 ± 17	61 ± 15	66 ± 13	69 ± 12	71 ± 11
Number of hospitalizations per year ^2^, mean ± SD		0.38 ± 0.87	0.21 ± 0.62	0.30 ± 0.76	0.44 ± 0.91	0.63 ± 1.06	1.00 ± 1.36
Patients with at least one hospitalization per year ^1^, *n* (%)		296,237 (24.8)	75,220 (15.8)	66,557 (21.3)	55,889 (29.3)	42,622 (38.9)	55,949 (53.1)
The average length of stay per year ^2^, mean ± SD		10.30 ± 13.74	7.88 ± 13.35	8.86 ± 12.62	10.03 ± 12.95	11.53 ± 13.39	14.62 ± 15.34
Length of stay per year ^1^, *n* (%)	1–3	98,274 (33.2)	36,155 (48.1)	26,053 (39.1)	17,005 (30.4)	10,101 (23.7)	8960 (16.0)
	4–6	47,723 (16.1)	12,554 (16.7)	11,202 (16.8)	9511 (17.0)	6875 (16.1)	7581 (13.5)
	7+	150,240 (50.7)	26,511 (35.2)	29,302 (44.0)	29,373 (52.6)	25,646 (60.2)	39,408 (70.4)
Number of rehospitalizations per year ^2^, mean ± SD		0.24 ± 0.82	0.17 ± 0.70	0.20 ± 0.80	0.23 ± 0.83	0.28 ± 0.84	0.38 ± 0.95
Patients with at least one rehospitalization per year ^1^, *n* (%)		44,122 (14.9)	7809 (10.4)	7951 (11.9)	7895 (14.1)	7466 (17.5)	13,001 (23.2)
Number of primary visits provided per year ^2^, mean ± SD		9.25 ± 7.32	6.74 ± 5.62	8.95 ± 6.43	10.81 ± 7.33	12.54 ± 8.07	15.30 ± 9.80
Number of specialist visits provided per year ^2^, mean ± SD		6.52 ± 7.14	4.32 ± 5.25	6.08 ± 6.40	7.88 ± 7.34	9.53 ± 8.22	12.23 ± 9.81

All *p*-values are less than 0.001. Tests used to compare patients across five groups (1, 2, 3, 4, 5+ chronic conditions): ^1^ Pearson’s Chi-squared test; ^2^ Kruskal–Wallis rank sum test. Continuous variables are presented as mean ± standard deviation or median (interquartile range). Categorical variables are expressed as *n* (%). Bolded text indicates statistical significance.

**Table 4:** Characteristics and healthcare resource utilization based on the complexity of multimorbidity.

Characteristics		Type of Multimorbidity			
		≥2 Chronic Conditions	Complex Multimorbidity	Type of Complex Multimorbidity	
				Complex Cardiac	Complex Non-Cardiac
Number of patients, *n* (%)		717,386 (31.0)	670,312 (28.9)	569,732 (24.6)	100,580 (4.3)
Gender ^1^, *n* (%)	Women	453,785 (63.3)	428,493 (63.9)	362,274 (63.6)	66,219 (65.8)
	Men	263,601 (36.7)	241,819 (36.1)	207,458 (36.4)	34,361 (34.2)
Age (year) ^2^, mean ± SD		65 ± 14	65 ± 14	67 ± 13	55 ± 15
Number of hospitalizations per year ^2^, mean ± SD		0.49 ± 0.98	0.50 ± 1.00	0.51 ± 1.00	0.42 ± 0.95
Patients with at least one hospitalization per year ^1^, *n* (%)		221,017 (30.8)	208,080 (31.0)	180,881 (31.7)	27,199 (27.0)
The average length of stay per year ^2^, mean ± SD		11.13 ± 13.77	11.25 ± 13.94	11.39 ± 13.76	10.33 ± 15.09
Length of stay per year ^1^, *n* (%)	1–3 days	62,119 (28.1)	58,348 (28.0)	48,205 (26.7)	10,143 (37.3)
	4–6 days	35,169 (15.9)	32,702 (15.7)	28,471 (15.7)	4231 (15.6)
	≥7 days	123,729 (56.0)	117,030 (56.2)	104,205 (57.6)	12,825 (47.2)
Number of rehospitalizations per year ^2^, mean ± SD		0.27 ± 0.86	0.28 ± 0.87	0.28 ± 0.86	0.28 ± 0.97
Patients with at least one rehospitalization ^1^ per year, *n* (%)		36,313 (16.4)	34,583 (16.6)	30,606 (16.9)	3977 (14.6)
Number of primary visits provided per year ^2^, mean ± SD		10.92 ± 7.83	11.10 ± 7.91	11.18 ± 7.88	10.64 ± 8.03
Number of specialist visits provided per year ^2^, mean ± SD		7.99 ± 7.82	8.22 ± 7.92	7.95 ± 7.84	9.76 ± 8.18

All *p*-values are less than 0.001. Tests used to compare complex cardiac vs. complex non-cardiac multimorbidity: ^1^ Pearson’s Chi-squared test; ^2^ Mann–Whitney U test. Continuous variables are presented as mean ± standard deviation or median (interquartile range). Categorical variables are expressed as *n* (%). Bolded text indicates statistical significance. *p*-value is given for the comparison of complex cardiac and complex non-cardiac patients.

**Table 5:** The hurdle negative binomial regression and truncated negative binomial models for healthcare utilization with multimorbidity group as a covariate.

Characteristics	Type of Multimorbidity (Ref. = Single Disease)	Univariate		Multivariate *	
		Count Part	Zero Part	Count Part	Zero Part
		IRR (95% CI) for the Number of Healthcare Utilization	OR (95% CI) for Having Healthcare Utilization	IRR (95% CI) for the Number of Healthcare Utilization	OR (95% CI) for Having Healthcare Utilization
Hospitalizations per year	Non-complex	**1.17 (1.12, 1.22)**	**2.02 (1.98, 2.06)**	1.08 (1.03, 1.13)	**1.79 (1.75, 1.83)**
	Complex cardiac	**1.85 (1.82, 1.89)**	**2.48 (2.46, 2.50)**	**1.75 (1.71, 1.79)**	**2.23 (2.21, 2.25)**
	Complex non-cardiac	**1.67 (1.62, 1.73)**	**1.98 (1.95, 2.01)**	**1.67 (1.62, 1.72)**	**1.96 (1.93, 1.99)**
Rehospitalizations per year	Non-complex	**0.47 (0.41, 0.53)**	**1.33 (1.26, 1.41)**	**0.49 (0.44, 0.56)**	**1.17 (1.11, 1.24)**
	Complex cardiac	1.04 (0.99, 1.10) ^#^	**1.76 (1.71, 1.81)**	1.12 (1.06, 1.19)	**1.60 (1.55, 1.64)**
	Complex non-cardiac	**1.52 (1.40, 1.64)**	**1.48 (1.42, 1.54)**	**1.56 (1.44, 1.69)**	**1.47 (1.41, 1.53)**
Primary visits provided per year	Non-complex	**1.25 (1.24, 1.26)**	**1.23 (1.26, 1.31)**	**1.34 (1.33, 1.34)**	**1.58 (1.48, 1.69)**
	Complex cardiac	**1.66 (1.66, 1.67)**	**3.49 (3.37, 3.61)**	**1.75 (1.75, 1.76)**	**4.24 (4.09, 4.39)**
	Complex non-cardiac	**1.58 (1.57, 1.59)**	**3.46 (3.22, 3.72)**	**1.58 (1.57, 1.59)**	**3.45 (3.21, 3.71)**
Specialist visits provided per year	Non-complex	1.04 (1.03, 1.05)	**1.25 (1.22, 1.28)**	1.11 (1.10, 1.12)	**1.75 (1.71, 1.80)**
	Complex cardiac	**1.67 (1.67, 1.68)**	**2.42 (2.40, 2.45)**	**1.75 (1.74, 1.76)**	**3.30 (3.27, 3.34)**
	Complex non-cardiac	**1.94 (1.93, 1.95)**	**5.58 (5.42, 5.73)**	**1.94 (1.92, 1.95)**	**5.89 (5.73, 6.05)**
Length of stay per year	Non-complex	**1.22 (1.18, 1.25)**		1.09 (1.06, 1.13)	
	Complex cardiac	**1.56 (1.54, 1.58)**		**1.43 (1.41, 1.45)**	
	Complex non-cardiac	**1.39 (1.36, 1.42)**		**1.38 (1.35, 1.40)**	

*—adjusted by age and sex, ^#^—*p* = 0.142. All *p*-values are less than 0.001, unless otherwise specified. Bolded values indicate where the lower confidence limit exceeds 1.10 or, for ratios less than 1, where the upper confidence limit is below 0.90. Ref., reference group; IRR, Incidence Rate Ratio; OR, Odds Ratio; CI, Confidence Interval.

## Data Availability

The data from this study are available from the Lithuania National Health Insurance Fund, but restrictions apply to the availability of these data, which were used under license for the current study and are not publicly available. The data are however available from the authors upon reasonable request and with the permission of the Lithuania National Health Insurance Fund.

## Supplementary Materials

The following supporting information can be downloaded at: https://www.mdpi.com/article/10.3390/jcdd12020047/s1, Table S1: The hurdle negative binomial regression and truncated negative binomial models for healthcare utilization with number of diseases as covariate.
