# Supplementary material for: Prevalence of Multimorbidity in Lithuania: Insights from National Health Insurance Fund Data
Source: J Cardiovasc Dev Dis. 2025 Jan 26;12(2):47. doi: 10.3390/jcdd12020047 (PMC11856744; doi:10.3390/jcdd12020047)
Supplement: Supplementary file 1 [file jcdd-12-00047-s001.zip › jcdd-3426627-supplementary.pdf]

Table S1. The hurdle negative binomial regression and truncated negative binomial models for healthcare utilization with number of diseases as covariate.

Characteristics	Number of diseases (ref. = 1)	Univariate		Multivariate*	
		Count part	Zero part	Count part	Zero part
		IRR (95% CI) for the number of healthcare utilization	OR (95% CI) for having healthcare utilization	IRR (95% CI) for the number of healthcare utilization	OR (95% CI) for having healthcare utilization
Hospitalizations per year	2	**1.24 (1.21, 1.27)**	**1.45 (1.43, 1.46)**	**1.26 (1.23, 1.29)**	**1.42 (1.41, 1.44)**
	3	**1.48 (1.45, 1.52)**	**2.21 (2.18, 2.24)**	**1.52 (1.48, 1.56)**	**2.15 (2.13, 2.18)**
	4	**1.83 (1.78, 1.87)**	**3.40 (3.35, 3.45)**	**1.90 (1.84, 1.95)**	**3.30 (3.25, 3.35)**
	5+	**2.75 (2.68, 2.81)**	**6.04 (5.95, 6.13)**	**2.89 (2.82, 2.97)**	**5.84 (5.75, 5.93)**
Rehospitalizations per year	2	1.15 (1.08, 1.23)	**1.17 (1.13, 1.21)**	**1.22 (1.14, 1.31)**	**1.15 (1.11, 1.19)**
	3	1.08 (1.01, 1.16)	**1.42 (1.37, 1.47)**	1.18 (1.10, 1.26)	**1.39 (1.34, 1.44)**
	4	0.95 (0.89, 1.02)[#]	**1.83 (1.77, 1.90)**	1.06 (0.99, 1.14)[$]	**1.80 (1.73, 1.87)**
	5+	1.06 (1.00, 1.12)[†]	**2.61 (2.53, 2.69)**	**1.23 (1.15, 1.31)**	**2.58 (2.50, 2.67)**
Primary visits provided per year	2	**1.32 (1.32, 1.33)**	**2.67 (2.57, 2.78)**	**1.39 (1.39, 1.39)**	**3.04 (2.92, 3.16)**
	3	**1.60 (1.60, 1.61)**	**3.11 (2.95, 3.27)**	**1.73 (1.72, 1.74)**	**3.77 (3.58, 3.97)**
	4	**1.87 (1.86, 1.87)**	**3.38 (3.16, 3.62)**	**2.05 (2.04, 2.06)**	**4.29 (4.00, 4.59)**
	5+	**2.28 (2.27, 2.28)**	**5.34 (4.90, 5.82)**	**2.53 (2.52, 2.55)**	**7.00 (6.42, 7.64)**
Specialist visits provided per year	2	**1.30 (1.30, 1.31)**	**1.74 (1.72, 1.76)**	**1.38 (1.38, 1.39)**	**2.20 (2.18, 2.23)**
	3	**1.62 (1.61, 1.63)**	**2.83 (2.78, 2.87)**	**1.77 (1.76, 1.78)**	**4.03 (3.97, 4.10)**
	4	**1.93 (1.92, 1.95)**	**3.86 (3.77, 3.95)**	**2.15 (2.14, 2.17)**	**5.94 (5.80, 6.08)**
	5+	**2.45 (2.43, 2.47)**	**5.97 (5.81, 6.14)**	**2.78 (2.76, 2.80)**	**9.79 (9.52, 10.1)**
Length of stay per year	2	**1.15 (1.14, 1.17)**		**1.14 (1.12, 1.16)**	
	3	**1.34 (1.32, 1.36)**		**1.31 (1.29, 1.33)**	
	4	**1.58 (1.56, 1.61)**		**1.54 (1.52, 1.57)**	
	5+	**2.09 (2.05, 2.12)**		**2.04 (2.00, 2.08)**	

*-adjusted by age and sex; # - p = 0.135; † - p = 0.054; $ - p = 0.103.

All p-values are less than 0.001, unless otherwise specified. Bolded values indicate where the lower confidence limit exceeds 1.10 or, for ratios less than 1, where the upper confidence limit is below 0.90. Ref., reference group; IRR, Incidence Rate Ratio; OR, Odds Ratio; CI, Confidence Interval.
